# Collaborative driving style classification method enabled by majority voting ensemble learning for enhancing classification performance

**DOI:** 10.1371/journal.pone.0254047

**Published:** 2021-07-19

**Authors:** Yi Guo, Xiaolan Wang, Yongmao Huang, Liang Xu

**Affiliations:** School of Electrical and Electronic Information, Xihua University, Chengdu, China; Vellore Institute of Technology: VIT University, INDIA

## Abstract

The classification of driving styles plays a fundamental role in evaluating drivers’ driving behaviors, which is of great significance to traffic safety. However, it still suffers from various challenges, including the insufficient accuracy of the model, the large amount of training parameters, the instability of classification results, and some others. To evaluate the driving behaviors accurately and efficiently, and to study the differences of driving behaviors among various vehicle drivers, a collaborative driving style classification method, which is enabled by ensemble learning and divided into pre-classification and classification, is proposed in this paper. In the pre-classification process, various clustering algorithms are utilized compositely to label some typical initial data with specific labels as aggressive, stable and conservative. Then, in the classification process, other unlabeled data can be classified accurately and efficiently by the majority voting ensemble learning method incorporating three different conventional classifiers. The availability and efficiency of the proposed method are demonstrated through some simulation experiments, in which the proposed collaborative classification method achieves quite good and stable performance on driving style classification. Particularly, compared with some other similar classification methods, the evaluation indicators of the proposed method, including accuracy, precision, recall and F-measure, are improved by 1.49%, 2.90%, 5.32% and 4.49% respectively, making it the best overall performance. Therefore, the proposed method is much preferred for the autonomous driving and usage-based insurance.

## Introduction

The occurrence of traffic accidents was directly related to the driver’s operation, and bad driving styles lead to more traffic accidents [1]. According to data released by China’s Ministry of Public Security, accident deaths caused by improper driving account for 88.91% of all deaths [2]. Similarly, data released by the National Highway Traffic Safety Administration (NHTSA) shows that subjective driver error is responsible for 94% of all crashes [3]. Therefore, an effective driving style classification model is of great significance for driving data analysis and traffic safety. Analysis of driving behaviors and classification of driving styles not only can prevent traffic accidents effectively, but also can be applied to human-centric vehicle control systems [4], intelligent transportation systems [5], and power management for electric vehicles [6]. A driving style classification model, which can classify driving styles more efficiently is proposed in this paper.

**Fig 1:**
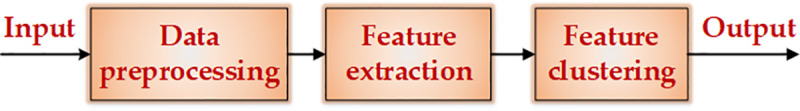
Framework scheme of clustering-based driving style classification method.

**Fig 2:**
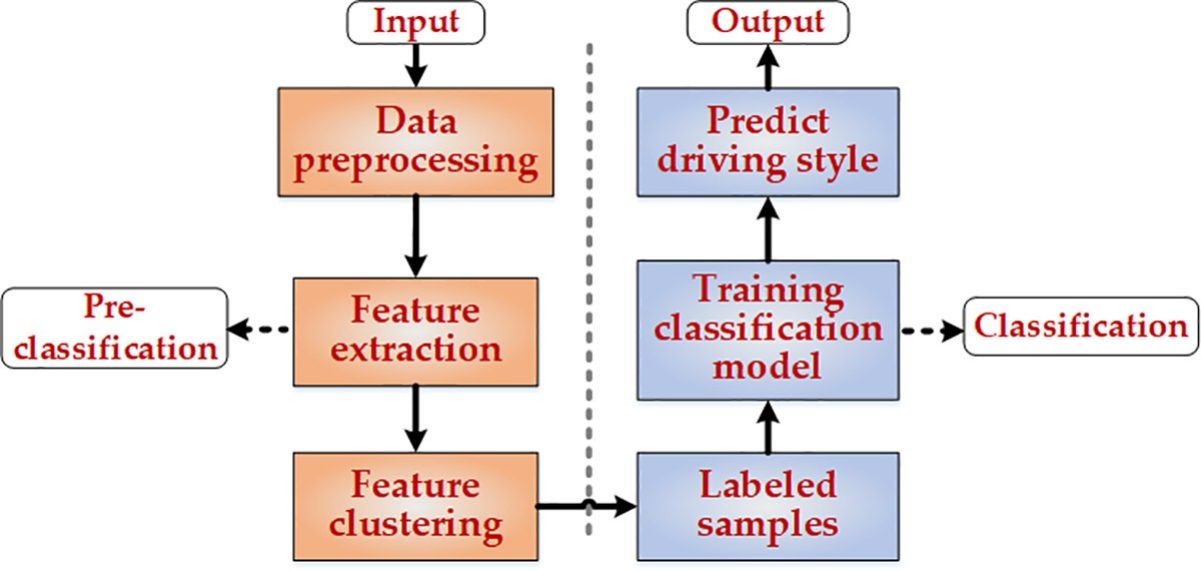
Framework scheme of driving style classification method based on clustering and classification.

The driving style mirrors the driver’s personalized vehicle operation mode, including driving speed, concentration level, vehicle control strategy, and so on, which can well reflect the driver’s individual driving characteristics [7, 8]. Lots of approaches had been studied to recognize driving style of unlabeled data in previous research, which could be roughly categorized into two groups: clustering-based and classification-based. Clustering-based methods mainly include K-means [9, 10], The density-based spatial clustering of applications with noise (DBSCAN) [11], agglomerative hierarchy [12], fuzzy c-means (FCM) [13], and some others. For example, in term of 10 driving characteristics, K-means algorithm was applied to classify drivers into calm type, normal type and aggressive type by applying in [9]. The framework of clustering-based was shown in Fig 1. The research method based on classification was a more in-depth study based on clustering method, the framework of it was shown in Fig 2. In this framework, the results of cluster analysis were used to train and test the classification algorithm. The main classification methods include various neural networks [14–18], decision tree [19], random forest (RF) [20], extreme gradient boosting (XGB) [21], support vector machine (SVM) [22, 23], Bayes classifier [24, 25], AdaBoost [26], and Dempster-Shafer (D-S) evidence theory [27]. Driving style classification could be realized by conventional methods, but there were still many limitations: (1) The clustering-based methods have a problem of re-cluster analysis for newly added data, as new data were generated, it needed to re-analyze the whole data set. (2) Bayes and neural networks belong to the conventional statistical learning classification methods. A large number of training samples were required for them. The larger the number of samples, the closer the test results will be to the real results, which was rare in practical application. (3) Although the decision tree and SVM were suitable for classifying small number samples, the results of a single classifier were unstable and the model was easy to fall into overfitting. Generally speaking, the conventional driving style classification models were generally plagued by low accuracy rate of classifications, poor robustness of models, high complexity of algorithms, and single evaluation indicator of results. In solving the above problems, ensemble learning had a good performance, and it has been proved widely in other fields [28, 29]. In the field of vehicle driving safety, an ensemble method based on CNN and GRU was proved that it could increases the detection performance of attack detection and subsequently brings about improved detection performance [30]. To enhance the efficiency and accuracy of the classification models, a collaborative driving style classification method enabled by majority voting ensemble learning was proposed in this paper. It was enabled by ensemble learning and divided into pre-classification and classification. The ensemble learning was adopted in both pre-classification and classification processes. In the pre-classification process, FCM and spectral clustering (SC) were utilized to initially classify the driving behavior data and labeled some typical initial data with specific labels as aggressive, stable and conservative. Then, the labeled data were used to train the classification algorithms. Finally, the majority voting ensemble method incorporating classification and regression tree (CART), SVM, and K-nearest neighbor (KNN) were used to classify other unlabeled data. Compared with other ensemble learning methods, such as RF and AdaBoost, the proposed method’s accuracy, precision, recall and F-measure were improved by 1.49%, 2.90%, 5.32% and 4.49% respectively. What’s more, the proposed method had better generalization performance and efficiency than some other machine learning methods under the same data set.

The remainder of this paper is structured as follows: Section II introduces some theoretical background. The majority voting ensemble method is detailed in Section III, following with the experiment results in Section IV. Section V discusses the results, with the conclusions given finally.

## Guidelines for manuscript preparation

### Evaluation indicator

In the final evaluation, we comprehensively evaluate the model from two aspects: the rationality of the classification results and the validity of the classifier. Comprehensive evaluation of the classification results’ rationality is carried out by introducing some clustering evaluation indicators, including internal and external effectiveness indicators. The internal effectiveness indicator is mainly based on the structural information of the data set, and the cluster partition is evaluated from the aspects of compactness, separability, connectivity and overlap. The external effective indicator means that when the external information of the data set is available, the performance of different clustering algorithms can be evaluated by comparing the matching degree between clustering division and the external criteria. Moreover, two internal validity indicators, namely the Davies-Bouldin index [31] and Calinski-Harabasz index [32] are utilized to evaluate the results here owing to the data used for experiment being unlabeled.

The Davies-Bouldin index uses the distance from the sample point in the cluster to its cluster center to estimate the tightness within the cluster, and the distance between the cluster centers represents the separation between clusters. The smaller the Davies-Bouldin Index is, the better the classification effect is. Davies-Bouldin Index is defined as:

$$DBI=\frac{1}{k}\sum_{i=1}^{k}R_{i}$$ (1)

Where *k* is the number of clusters and *R*_*i*_ is defined as:

$$R_{i}=\underset{i\neq j}{\max}R_{ij}$$ (2)

Where *R*_*ij*_ is the similarity measure between cluster *C*_*i*_ and *C*_*j*_, and is defined as:

$$R_{ij}=\frac{S_{i}+S_{j}}{\left|m_{i}-m_{j}\right|}$$ (3)

Where *S*_*i*_ is the standard error between the sample point and *m*_*i*_ in the *C*_*i*_. and is defined as:

$$S_{i}=\sqrt{\frac{1}{\left|C_{i}\right|}\sum_{x\in C_{i}}{\left|x-m_{i}\right|}^{2}}$$ (4)

Where |*C*_*i*_| is the number of data in cluster *C*_*i*_ and *m*_*i*_ is the center of *C*_*i*_.

The Calinski-Harabasz index measures the tightness of the cluster by calculating the square sum of the distance between each point in the cluster and the center of the cluster, and measures the separation of the data set by calculating the square sum of the distance between various centers and the data center point. The Calinski-Harabasz is the ratio of the distance between the clusters to the distance within one cluster [33]. The Calinski-Harabasz index is defined as:

$$\mathrm{CH}=\frac{T_{r}(S_{B})/\left(K-1\right)}{T_{r}(S_{w})/\left(n-K\right)}$$ (5)

Where *n* is the number of clusters, *K* is the current cluster, and the *T*_*r*_ (*S*_*B*_) and *T*_*r*_ (*S*_*w*_) are defined as:

$$T_{r}(S_{B})=\sum_{i=1}^{K}n_{i}\times d(v_{i},v)$$ (6)

$$T_{r}(S_{w})=\sum_{i=1}^{K}\sum_{j=1}^{n}d(x_{j},v_{i})$$ (7)

*T*_*r*_ (*S*_*B*_) is the trace of the dispersion matrix between clusters, *T*_*r*_ (*S*_*w*_) is the trace of the dispersion matrix within the cluster, *n*_*i*_ represents the number of clusters, *K* represents the current class, and *v* represents the center of mass of the entire dataset, *v*_*i*_ represents the center of *class-i*. The larger the Calinski-Harabasz index is, the closer the cluster itself is and the more dispersed it is between clusters, which is the better clustering result.

To compare the effectiveness of the classifiers, accuracy, precision, recall and F-measure are used to evaluate the classification model. The indexes are defined as:

$$\mathrm{Accuracy}=\frac{TP+TN}{TP+TN+FP+FN}$$ (8)

$$\mathrm{Precision}=\frac{TP}{TP+FP}$$ (9)

$$\mathrm{Recall}=\frac{TP}{TP+FN}$$ (10)

$$F‐\mathrm{measure}=\frac{2\times Precision\times Recall}{Precision+Recall}$$ (11)

Where TP is the number of samples belonging to Class C that were correctly classified into class C. FP is the number of samples that did not belong to Class C that were misclassified into class C. TN is the number of samples that do not belong to category C and are correctly classified into other classes. FN is the number of samples belonging to category C that were misclassified into other classes.

### Driving style classification model based on majority voting ensemble learning

**Fig 3:**
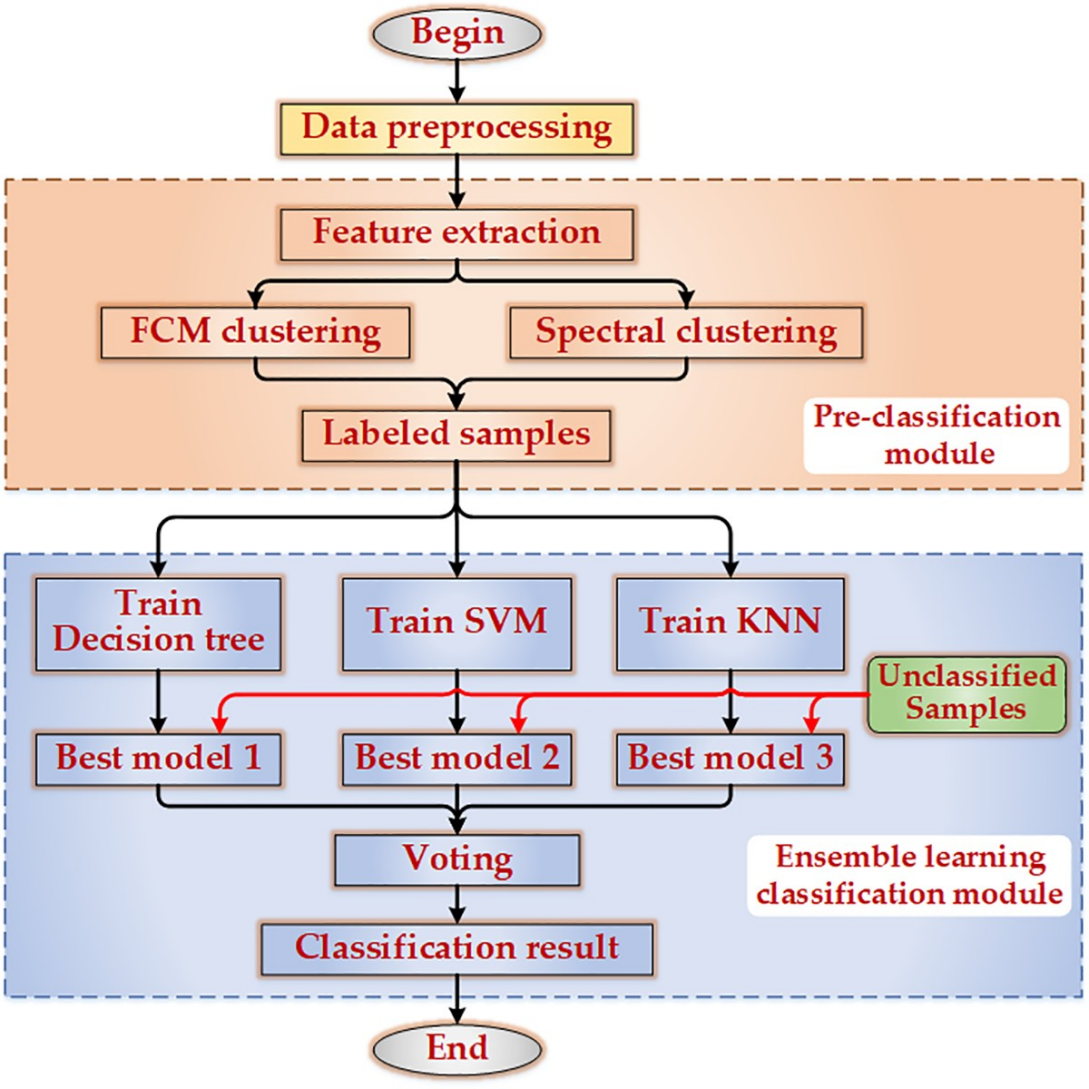
Driving style classification model flow based on ensemble learning.

For strengthening the generalization ability, accuracy, and stability, some improvements have been made to model 2 in Fig 2. The main improvement is that the majority voting ensemble learning is utilized in feature clustering and classification model, which changes the way of pre-classification and classification of the original model using a single algorithm. For pre-classification module, typical samples were labeled as aggressive, stable and conservative through two clustering algorithms. After experimental comparison, FCM and spectral clustering were used to classify and label the data initially. By comparing the clustering results of the two methods, the same results are labeled as typical samples, the rest data are retained as unclassified data. For classification module, excellent generalization ability, stability, and accuracy are reached by classifying the unclassified data in a manner of majority voting ensemble learning. Through theoretical comparison and experimental analysis, CART, SVM, and KNN are selected for the majority voting ensemble learning. The flow of the model is shown in Fig 3.

The main steps of the driving style classification model based on majority voting ensemble learning are as follows.

(1) Data pre-processing: The useless attributes, and the noise data of the 450 transport vehicles are removed, and the missing data are filled with some filling regular. Moreover, the trip of each vehicle is divided into micro-trips.

(2) Pre-classification module:

1) Feature extracting: Based on the pre-processed data, the feature parameters of driving behavior are extracted by using the identification method of bad driving behavior;

2) Feature clustering: Determining the number of clusters, and clustering the feature parameters with the FCM and SC;

3) Data labeling: The samples with the same clustering result of the two clustering algorithms are labeled with specific labels as aggressive, stable and conservative, and the other unlabeled data are taken as unclassified samples for subsequent reclassification.

(3) Classification module:

1) Training individual classifiers: Using labeled data as training dataset to train CART, SVM, and KNN model;

2) Classification of unclassified data: The trained CART model, SVM model, and KNN model are used to classify the unclassified data;

3) Ensemble decision: Combining the three models’ classification results by majority voting, thereby realizing the evaluation of the driving style of each driver.

### Data preparation

The dataset of question C of the “2018 Teddy Cup Data Mining Challenge” was adopted for the experiment in this paper. The details of the dataset are shown in Table 1. The dataset includes 13 attributes of 450 transportation vehicles, such as vehicle number, latitude, and longitude coordinates, direction angle, ignition status, mileage, instantaneous speed, acquisition time, and some others. Attributes and their meanings of the data are presented in Table 2. Among them, the value of the four attributes of the left-turning and right-turning signals and the handbrake and footbrake is always 0, which regard as invalid attributes. The total number of data reached 25 million.

**Table 1:** The details of the dataset.

Data source	2018 Teddy Cup Data Mining Challenge
Whether open source	Yes
Feature dimension	13
Number of vehicles	450
Amount of data	25000000

**Table 2:** Data attributes and their meanings.

Attribute name	Explanation
vehicleplatenumber	Vehicle ID
device_num	Number of the device that collects data
direction_angle	Direction angle of the vehicle
lng	Longitude of the vehicle
lat	Latitude of the vehicle
acc_state	The status of the engine
location_time	The moment of data collection
gps_speed	Instantaneous velocity of a vehicle
mileage	The total mileage of the vehicle
right_turn_signals	Status of right-turning signal
left_turn_signals	Status of left-turning signal
hand_brake	Status of handbrake
foot_brake	Status of footbrake

The preprocessing procedures are shown in Table 3. Attribute specification, missing value filling, outlier correction and other operations were carried in the data preprocessing. Unavailable attributes should be deleted, partial missing mileage should be filled with average value of before and after value, and abnormal longitude and latitude should be replaced.

**Table 3:** Data preprocessing procedure.

Input: The original data D
/*Step1: Data reduction */
1) Remove useless attributes;
/*Step2: Outlier handling*/
2) Abnormal mileage deleted;
3) Zero speed filling;
4) Anomalous latitude and longitude correction;
/*Step3: Micro-trip division*/
5) Remove the data of vehicle stop period and divide the journey into sections;
**Output:** Prepared data D’

### Extraction of driving behavior feature parameters

According to the relevant research foundation of vehicle driving behavior recognition and bad driving behavior detection [34], some methods of identifying bad driving behavior are utilized to extract the feature parameters. In extraction of feature parameters, eight bad driving behaviors including fatigue driving times, long idling hot car times, extra-long idle times, rapid lane changes times, rapid acceleration times, rapid deceleration times, coasting with engine off times, and overspeed time are extracted. The identification methods of various types of bad driving behavior are as follows:

(1) Fatigued driving behavior: Single fatigue driving event is defined as a single continuous driving event of more than 4 hours during, which a single rest time is less than 20 minutes. If the cumulative driving time exceeds 8 hours in a single day, it is the cumulative fatigue driving event.

Suppose the vehicle has a total of *n* trips in a day, and the start time of the *i-th* trip is *T*_*is*_ and the end time is *T*_*ie*_, where *i = (1*, *2*,*…*,*n)*. Then, according to (12)–(14), the time *T*_*i*_ of the *i* trip, the time interval △*T* between the *i* trip and the *i+1* trip, and the total travel time of the single day DT can be calculated. If *T*_*i*_ is greater than 4 hours and △*T* is less than 20 minutes, it is a single fatigue driving event; If DT is greater than 8 hours, it is a cumulative fatigue driving event.


$$T_{i}=T_{ie}-T_{is}$$ (12)

$$\Delta T=T_{(i+1)s}-T_{ie}$$ (13)

$$\mathrm{DT}=\sum_{i}^{n}T_{i}$$ (14)

(2) Rapid speed change: Define that the acceleration of the vehicle is greater than 3m/s^2^ as a rapid acceleration event, and the acceleration is lesser than -3m/s^2^ as a rapid deceleration event.

Suppose the speed of the vehicle is *v* the time increment is *△t* and calculate the acceleration A according to (15). If A≥3m/s^2^, we think it is a rapid acceleration behavior; if A≤-3m/s^2^, we think it is a rapid deceleration behavior.


$$A=\frac{v(t+\Delta t)-v(t)}{\Delta t}$$ (15)

(3) Rapid lane change: Suppose the speed of the vehicle is *v*, the direction angle at the moment *i* is *R*_*i*_, and the time is *t*_*i*_, then the lane change angular speed *V* of the vehicle can be calculated by (16). As V≥20*°/s*, recording *T*_*1*_ and the direction angle *R*_*1*_ of that moment. As V*≤*5*°/s*, record the time *T*_*2*_ and the direction angle *R*_*2*_ of that moment. According to formulas (17)–(18), calculate the lane change duration T and the direction angle deflection D when restoring the lane change. If T*≤10s* and D*≤5°*, it is judged as a sudden lane change behavior, and *T*_*1*_, *R*_*1*_, *T*_*2*_, *R*_*2*_ are reset as zero.


$$V_{i}=\frac{R_{i+1}-R_{i}}{t_{i+1}-t_{i}}$$ (16)

$$D=|R_{2}-R_{1}|$$ (17)

$$T=T_{2}-T_{1}$$ (18)

(4) Bad idle speed: When the vehicle is ignited and the speed is 0, it is an idle speed behavior. In this paper, the idle time of the bad idle warm-up behavior is defined as 2-10min, and the idle behavior over 10min is defined as an extra-long idle behavior.

Suppose the time to start idling as *t*_*s*_ and the time to end the idling as *t*_*e*_, then the idle time of the vehicle T can be calculated according to formula (19). If 2min≤T≤10min, it is judged as a bad idle warm-up behavior, and if T>10min it is judged as an extra-long idle behavior.


$$T=t_{e}-t_{s}$$ (19)

(5) Flameout slide: When the vehicle speed *v*≠0, the status of the engine is 0, and the duration exceeds 2s, it is judged as a flameout slide event.

(6) Overspeed: According to “City Standards for Urban Road Engineering Design” (CJJ37-2012) [35], three speed limit thresholds are defined for vehicle speed, which are 60km/h, 80km/h, and 120km/h, meaning is in turn: the boundary of low speed and high speed, the boundary of highways and city roads, the boundary of ultra-high speed in highway. If the speed of the vehicle exceeds the relevant thresholds, it is determined to be an overspeed behavior.

Calling Baidu API to detect the driving section of the vehicle, the current speed is set as *V*, and the speed threshold of the corresponding section of the driving vehicle is *V*_*max*_. If *V*>*V*_*max*_, then the vehicle is judged to be overspeeded and the overspeed time *T* is recorded.

After extracting each bad driving behavior, the feature parameters are extracted as below:

$${\mathrm{Rate}}_{i}=\frac{C_{i}}{M}\times 1000$$ (20)

$$\mathrm{OS}=\frac{T\_over}{T\_total}$$ (21)

Rate_*i*_ is the rate of vehicle bad driving behavior, *C*_*i*_ is the number of single bad driving behaviors, *i*∈(1,2,…,7), corresponding to vehicle fatigue driving times, bad idling warm-up times, extra-long idling times, rapid lane changes times, rapid acceleration times, rapid deceleration times, and flameout slide times, *M* is the total mileage of the vehicle. OS is the ratio of vehicle overspeed time, *T_over* is the overspeed time, and *T_total* is the total time the vehicle is running.

The feature parameters and their parameter units are shown in Table 4.

**Table 4:** Driving behavior feature parameters and their units.

Parameter	Unit
Fatigue driving rate (FDR)	Times / 1000 km
Long idling hot car rate (LIHR)	Times / 1000 km
Extra-long idle rate (EIR)	Times / 1000 km
Rapid lane change rate (RLCR)	Times / 1000 km
Rapid acceleration rate (RAR)	Times / 1000 km
Rapid deceleration rate (RDR)	Times / 1000 km
Coasting with engine off rate (CEOR)	Times / 1000 km
Overspeed time ratio (OTR)	-

### Pre-classification of driving behavior based on FCM and SC

In pre-classification, there are some processes for achieving the purpose of dividing vehicle data into labeled dataset and unclassified dataset. Firstly, cluster the vehicle data into ***k*** types by FCM and spectral clustering. In this process, set the category number ***k*** and suppose the clustering result of FCM be expressed as ***C_f*(*i*)**, the cluster center as ***m***_***j***_, and the clustering result of spectral clustering be expressed as ***C_s*(*i*)**, where *i* = 1, 2, …, *n*, ***n*** is the number of samples. Then, compare ***C_f*(*i*)** with ***C_s*(*i*)**, if ***C_f*(*i*)** = ***C_s*(*i*)**, the sample ***i*** is labeled with its type label and divided into labeled dataset **X**_***i***_. Otherwise, the sample ***i*** is divided into unclassified dataset **P**_***i***_. The pre-classification procedure is presented in Table 5.

**Table 5:** Pre-classification procedures.

**Input:** Feature dataset, Number of clusters ***k***
/*Step1: Clustering driving behavior based on FCM */
1) Set the value of ***k***, use FCM for clustering;
2) Return the clustering result ***C_f(i)*** and cluster center ***m***_***j***_;
/*Step2: Clustering driving behavior based on SC*/
3) Set the value of ***k***, the similarity matrix generation method is Radial Basis Function, and use SC for clustering;
4) Return the clustering result ***C_s(i)***;
/*Step3: Pre-Classification of Driving Behavior*/
5)If ***C_f(i)*** = ***C_s(i)***:
6) Divide sample ***i*** into labeled samples **X**_***i***_;
7)else divide sample ***i*** into unclassified samples **P**_***i***_;
8) Return **X** and **P**
**Output:** labeled samples **X**_***i***_ and unclassified samples **P**_***i***_

### Driving behavior classification based on majority voting ensemble learning

The driving behavior classification model based on majority voting ensemble learning integrates CART, SVM, and KNN model to classify driving behavior. On the basis of pre-classification, labeled samples are trained and tested on the classification model. 80% of the labeled samples are used as training dataset **X** and 20% of the labeled samples are used as by **X**, and then select the three models that perform best in test dataset **P**. Firstly, every individual classifier was trained this task to classify the unclassified dataset **P**. Finally, the individual classifiers are combined by the majority voting strategy and thus obtain the final classification results. The classification procedure is presented in Table 6. There are some classifier parameters that need to be set. The penalty coefficient “C” of SVM is set as “0.8”, the kernel function is set as “linear”, and the decision method “decision_function_shape” is set as “ovr”. The maximum depth of the CART decision tree “max_depth” is set as “6”, the minimum impurity reduction amount “min_inpurity_split” is set as “0.1”, the maximum number of leaf nodes “max_leaf_nodes” is set as “28”, and the minimum sample number of leaf nodes “min_samples_leaf” is set as “1”. KNN does not need to set any other parameters. In addition, the computational complexity of the proposed method is *O(MN)*.

**Table 6:** Classification procedures.

**Input:** Labeled samples set **X**, unclassified samples set **P**
/*Step1: Training of individual classifiers*/
1) Training CART model with labeled sample set **X**;
2) Training SVM model with labeled sample set **X**;
3) Training KNN model with labeled sample set **X**;
4) Return the best models of the three algorithms;
/*Step2: Classification of driving styles using ensemble learning*/
5) Use the trained CART model to classify the data set **P**;
7) Use the trained SVM model to classify the data set **P**;
8) Use the trained KNN model to classify the data set **P**;
9) Combine the classification results of three individual classifiers by voting.
**Output:** Results of driving style classification

## Experiment and analysis

### Experiment setup and procedures

As sketched in Fig 3, the experimental procedures mainly consist of four steps: (1) Data preprocessing; (2) Extraction of feature parameters; (3) The feature parameters are clustered by FCM and SC, and the data are divided into labeled data and unclassified data; (4) Use labeled data to train and test CART, SVM, and KNN models, then use the three models to classify the unclassified data, finally, combine the results of three models by majority voting.

The experimental environment configuration and platform details of this experiment are shown in Table 7. Python was used as the development language for the entire experiment, and PyCharm 2018 and Anaconda 3.0 were used as the development environment. The experimental computer’s central processing unit (CPU) is Intel core i5-3230M, 2.6GHz, random access memory (RAM) is 4GB and the operating system is Windows 10 64-bit.

**Table 7:** Experimental configuration.

Configuration	Experimental configuration
Operating system	Windows 10 64-bit
CPU	Intel core i5-3230M, 2.6GHz
RAM	4GB
Development environment	PyCharm 2018
	Anaconda 3.0
Development language	Python 3.6.1

### Experimental result and analysis

**Fig 4:**
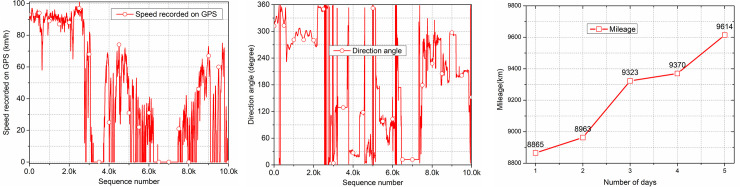
Data of AA00002. (a) Display of the first 10,000 speed data of the vehicle; (b) Display of the first 10,000 direction angle data of the vehicle; (c) Display of the vehicle mileage data.

**Fig 5:**
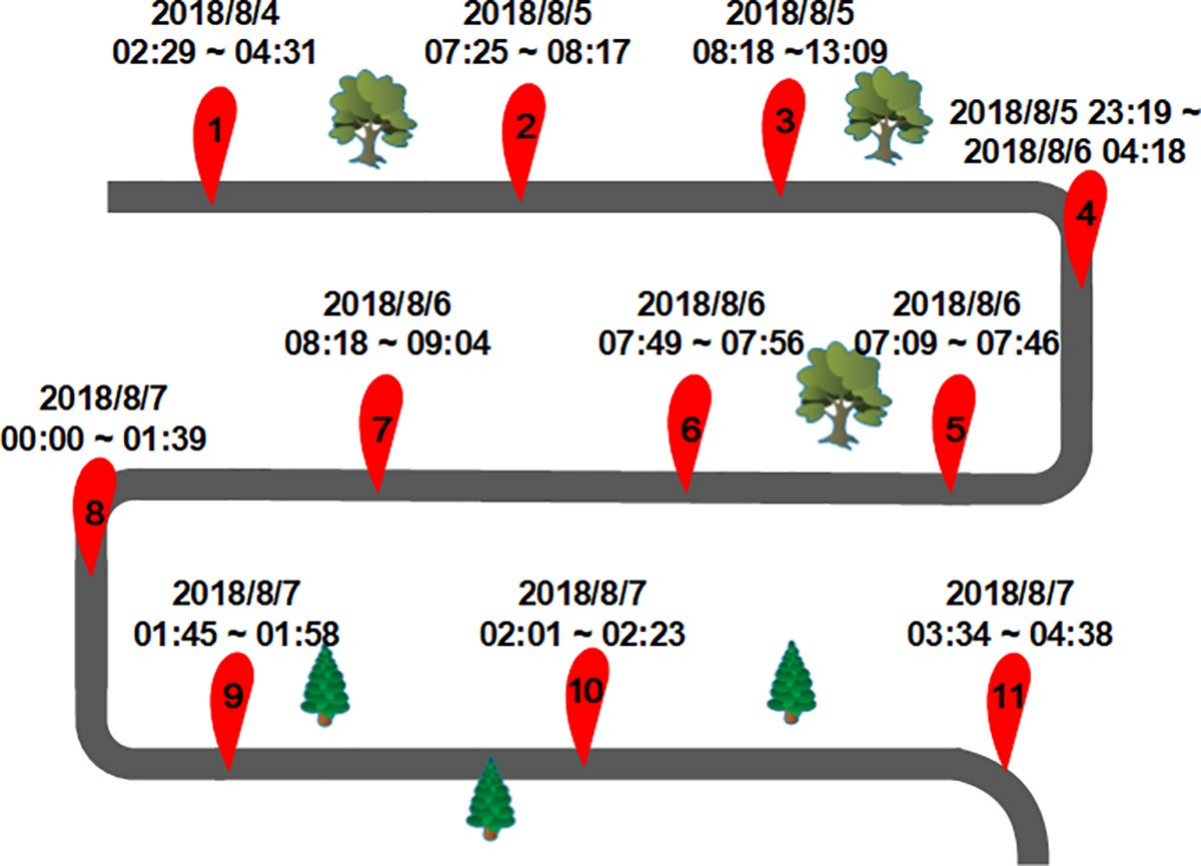
The result of micro-trip division of car AA00002.

First of all, in the preprocessing, all empty attributes are deleted, and only valid attributes are retained. When analyzing the data, it is found that there are abrupt mileage data and missing speed. For the abrupt mileage data, method of deleting the abrupt term is used for processing, and for the missing speed, mean filling is used. For abnormal longitude and latitude coordinates, angular velocity and traveling speed are used to predict the longitude and latitude coordinates of the next moment, and the predicted longitude and latitude coordinates are used to replace the abnormal longitude and latitude coordinates. Take car AA00002 as an example, the collected vehicle speed, and mileage are shown in Fig 4. Due to the large amount of data, we only show part of the data in the figure. And in the collected data, the starting mileage of the vehicle was 8,865 kilometers and the ending mileage was 9,614 kilometers, thus the vehicle traveled a total of 749 kilometers over 5 days. We can get the average speed of the vehicle by the vehicle mileage and travel time. Different attributes can be combined and verified with each other, thus the richer the data were, the more the accuracy of the detect driving events will be. At the same time, we divide the micro-trips of the vehicle trajectory data, and mark the data of all vehicles according to the micro-trip number. Take car AA00002 as an example, between August 4, 2018 and August 7, 2018, the vehicle made a total of 11 micro-trips, with the start and end times of each trip shown in Fig 5. As can be seen from the figure, the driving time of this vehicle is mostly concentrated at night, which conforms to the driving law of freight vehicles.

Secondly, according to the relevant research basis of vehicle driving behavior recognition and bad driving behavior detection mentioned in *“Extraction of driving behavior feature parameters”*, 450 transport vehicles were featured. The extraction results are shown in Table 8. The value of the overspeed time ratio is the result of magnifying the original value by 1000 times in Table 8. After features extraction, it was found that there are 3 vehicles have an empty data, so the data of 447 vehicles were finally put into use in the next stage.

**Table 8:** The results of feature parameters extraction.

FDR	LIHR	EIR	RLCR	RAR	RDR	CEOR	OTP
0.94	8.45	0.47	16.89	11.73	10.32	0	36.64
2.79	11.14	0	8.36	261.84	264.62	0	113.16
0	5.03	0	78.73	50.25	46.90	0	132.32
⋮	⋮	⋮	⋮	⋮	⋮	⋮	⋮

Thirdly, in pre-classification experiments, four clustering algorithms, i.e. K-means, DBSCAN, FCM and SC, are used for clustering. Finally, two algorithms with higher similarity of clustering results were selected as sub-algorithms of the pre-classification module. Experiment shows that the DBSCAN is a clustering algorithm based on density division, which has a good effect on the detection of noise points. However, the cluster radius and density thresholds need to be set in advance, which is not applicable to the dataset in this paper. And compared with FCM and spectral clustering, the clustering results of K- means have more differentiated samples, which can’t label the data as many as possible. Therefore, FCM and spectral clustering are selected as sub-algorithms in this paper. In the experiment, the value of cluster number is set as 3, FCM and SC cluster the feature parameters data respectively. After clustering by FCM and spectral clustering, the clustering results and initial classification results obtained are shown in Table 9. In the results of FCM, the number of samples classified as Type-I is 98, the number of samples classified as Type-II is 23, and the number of samples classified as Type-III is 327. In the results of spectral clustering, the number of samples classified as Type-I, Type-II and Type-III are 105, 24 and 319. It can be seen that the clustering results of the two clustering methods are generally consistent, which also shows the effectiveness of clustering. Comparing the clustering results of the two algorithms, the number of samples classified as Type-I by both methods is 94, the number of samples classified as Type-II by both methods is 23, and the number of samples classified as Type-III by both methods is 316. Therefore, there are 433 samples labeled as training data. And the number of unclassified samples is 14, which will be classified in the subsequent experimental steps. What’s more, from the clustering results, the number of the Type-I and Type-III occupies the vast majority of the entire dataset. This means that the majority of the company’s drivers behave well.

**Table 9:** Clustering results.

Method	Type I (Stable)	Type II (Aggressive)	Type III (Conservative)	Unclassified
FCM	98	23	327	--
SC	105	24	319	--
Final	94	23	316	14

**Fig 6:**
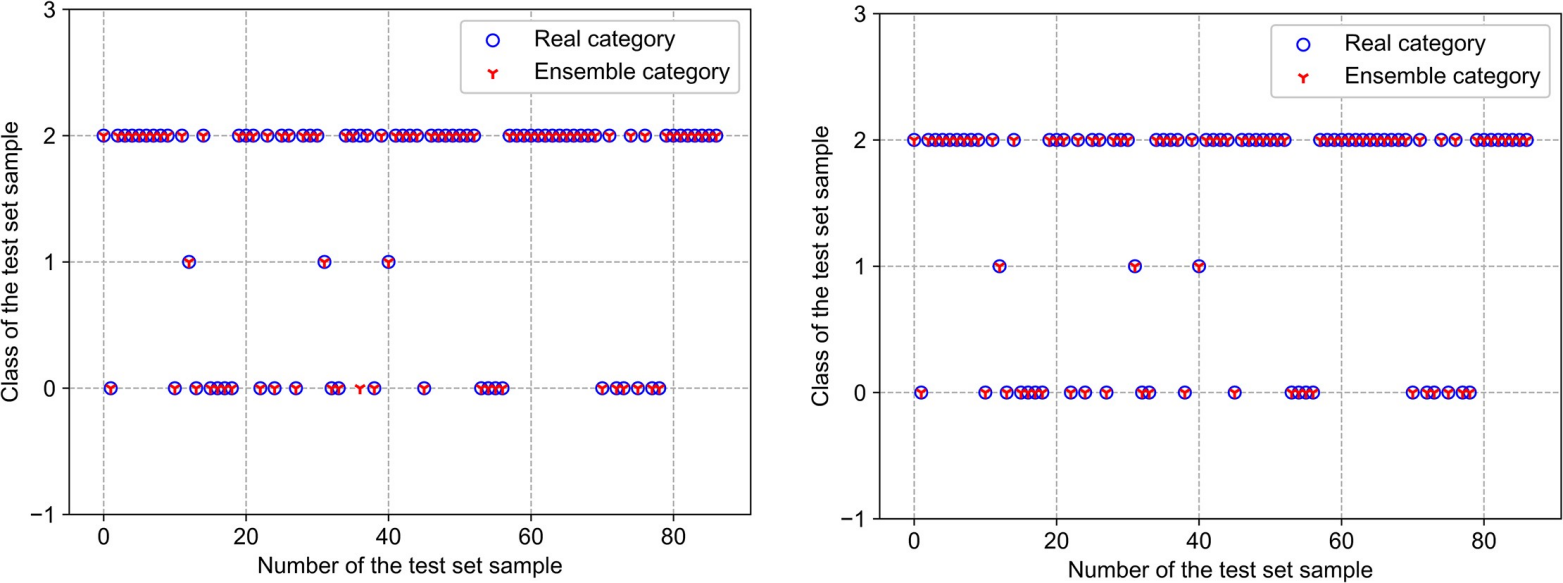
Classification results of test set sample by utilizing the proposed model. (a) Driving style recognition result with accuracy = 98.85%; (b) Driving style recognition result with accuracy = 100%.

Finally, according to the relevant theoretical basis in “*Driving behavior classification based on majority voting ensemble learning*”, the driving behavior classification model of majority voting ensemble learning is used to classify the unclassified samples. Firstly, each individual classifier is trained and tested by labeled dataset. Then, the results of the three classifiers are combined by majority voting to get a more stable classifier. The 433 labeled samples were divided into two parts. 80% of the samples were used as training dataset to train the classifiers, and 20% of the samples were used as test dataset to test the performance of the classifiers. With 346 random training samples and 87 test samples, the prediction accuracy of proposed model was fluctuated between 98.85% and 100%. The results of the model on the test dataset were shown in Fig 6. As can be seen from Fig 6, 87 test samples were classified, and the maximum number of samples with classification error was one, which demonstrated the excellent performance of the proposed method.

**Fig 7:**
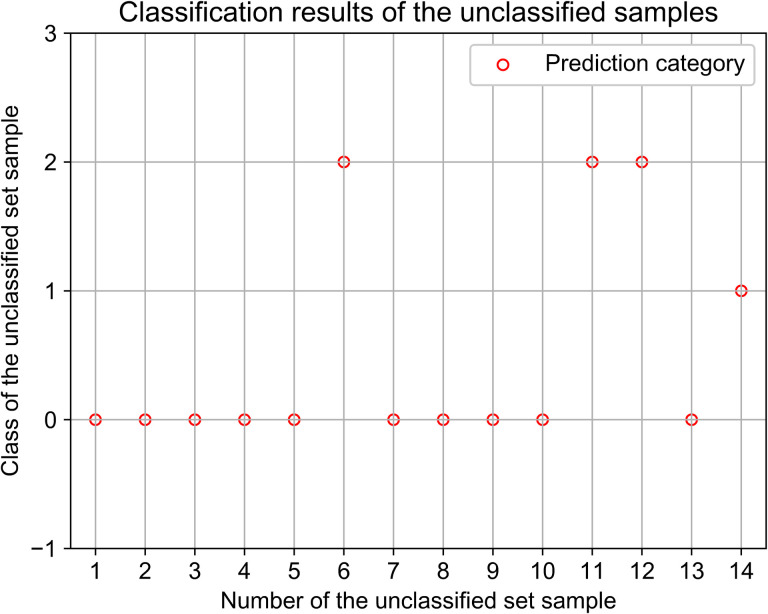
Classification results of unclassified samples.

In addition, three models with the best performance of individual classifiers were selected to predict the unclassified samples, the final classification results are shown in Fig 7. The specific data features and classification results are shown in Table 10. Where 10 of the 14 vehicles were classified as stable, 3 as conservative and 1 as aggressive. It can be seen from the table that the rapid acceleration and deceleration behaviors of vehicles classified as aggressive are significantly higher than those of other vehicles, which conforms to the driving characteristics of aggressive driving. Drivers classified as stable and conservative type have a small amount of bad driving behaviors, and less rapid acceleration and rapid deceleration. Moreover, drivers with severe acceleration and deceleration. behaviors also generally have rapid lane change behavior, and drivers without classification do not have coasting with engine off behavior.

**Table 10:** Classification results of unclassified samples.

Car ID	FDR	LIHR	EIR	RLCR	RAR	RDR	CEOR	OTP	Result	Style
AA00052	0	1.16	2.32	6.95	192.35	192.35	0	217.16	0	Stable
AA00154	0	19.91	0	9.19	199.08	194.49	0	79.51	0	Stable
AA00345	0	277.78	0	833.33	222.22	222.22	0	0	0	Stable
AB00465	1.03	1.03	2.06	20.64	62.95	288.96	0	71.61	0	Stable
AD00027	0.92	5.54	0	11.53	189.11	184.04	0	56.02	0	Stable
AD00050	1.76	2.34	0	2.93	146.87	145.11	0	125.74	2	Conservative
AD00130	0	38.27	2.55	413.27	260.20	283.16	0	52.87	0	Stable
AD00150	0	16.52	0	15.02	201.95	197.45	0	94.68	0	Stable
AD00163	0	9.64	0	23.66	198.07	201.58	0	62.05	0	Stable
AD00178	0	19.01	0	44.35	177.40	181.62	0	83.42	0	Stable
AD00267	1.68	3.35	0	1.68	136.87	130.73	0	159.94	2	Conservative
AD00292	2.33	4.08	0	4.66	167.35	167.93	0	108.57	2	Conservative
AD00344	2.05	0	0.68	7.51	197.95	184.30	0	135.35	0	Stable
AF00066	0	11.57	0	76.03	563.64	591.74	0	0	1	Aggressive

**Fig 8:**
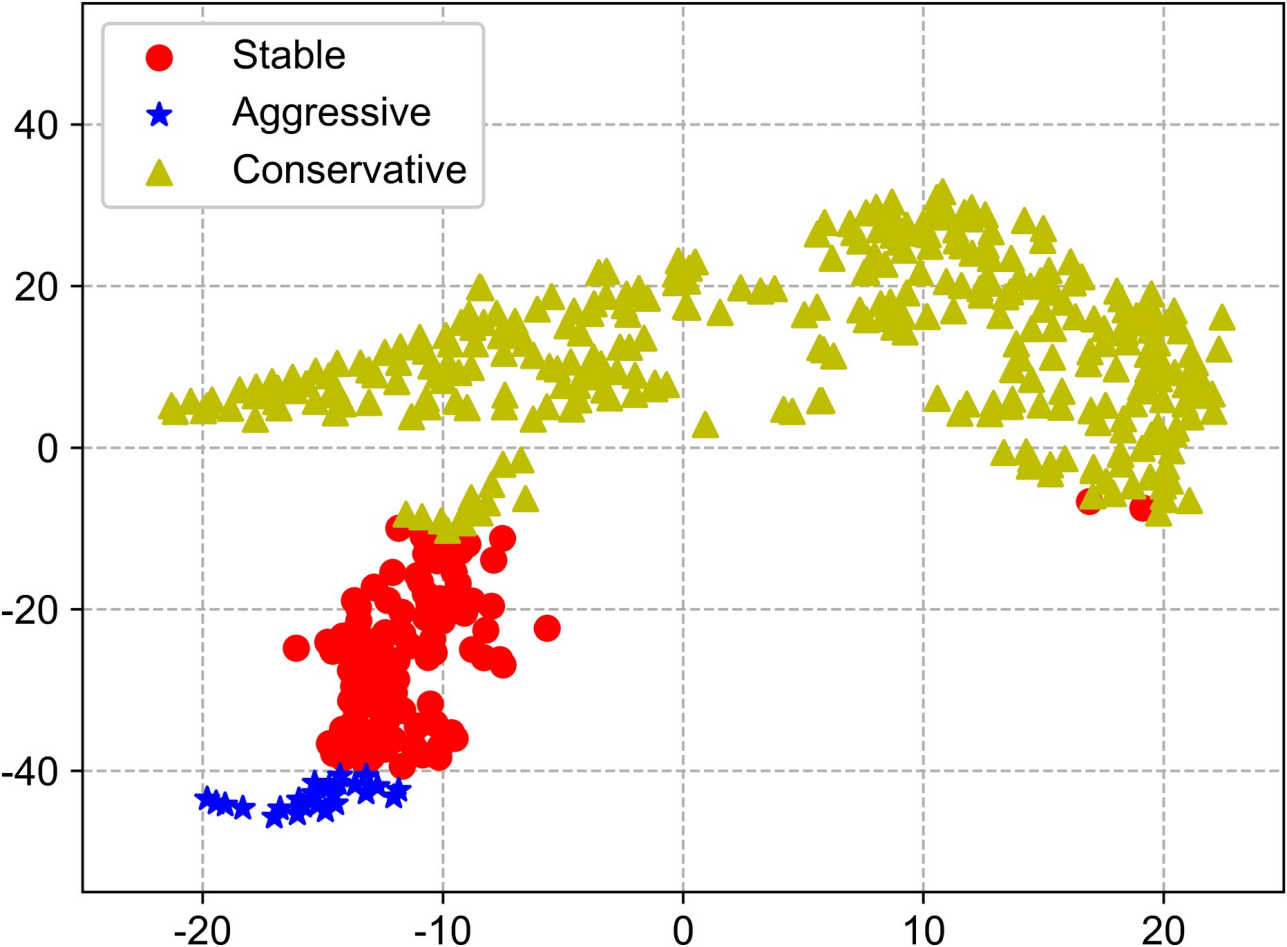
Visualization of clustering and classification results.

Subsequently, according to the final classification results, the relevant feature parameters, and the category labels of the training dataset, the driving style of the drivers can be classified as aggressive, stable and conservative type. T-distributed stochastic neighbor embedding (TSNE) was used to reduce the dimension of the data, and the classification results after dimension reduction were shown in Fig 8. It can be obtained from Fig 8 that 447 vehicles are effectively divided into 3 types, and the boundaries between the types are obvious. The yellow triangle samples are conservative driving style, which corresponds to label 2; the red dot samples are stable driving style, which corresponds to label 0; the blue star samples are aggressive driving style, which corresponds to label 1.

**Fig 9:**
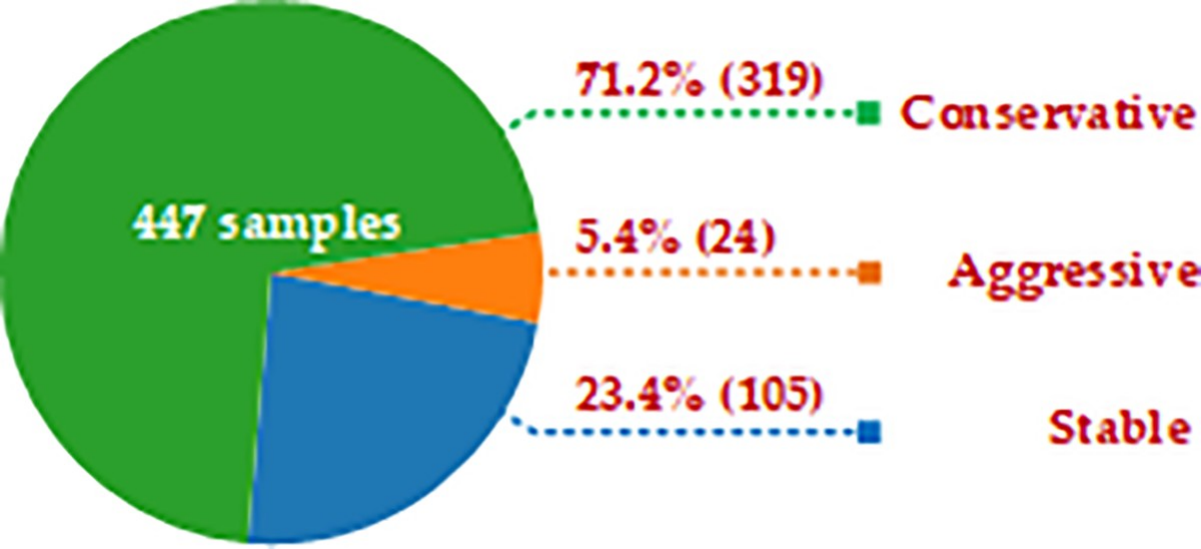
Driving style statistics results.

Thereafter, according to statistics, the driving style classification results of 447 drivers are shown in Fig 9. There are 105 drivers with stable driving style, accounting for 23.4% of the total dataset, 24 drivers with aggressive driving style, accounting for 5.4% of the total dataset, and 319 drivers with conservative driving style, accounting for 71.2% of the total data set. It can be seen that most of the drivers of this company are in compliance with the standard driving behavior and their driving style are more stable or conservative. A few drivers have more aggressive driving style. Drivers with aggressive driving style should be specially managed to regulate their driving behaviors and prevent the occurrence of driving accidents.

## Comparison and discussion

The validity of the classification results and the classification model are evaluated respectively in this work. The validity of the classification results was evaluated by internal cluster evaluation indicators Davies-Bouldin index and Calinski-Harabasz index, and the validity of the classification model was evaluated by accuracy, recall, precision and F-measure. The internal cluster evaluation indicator can effectively evaluate whether the classification is reasonable or not. The evaluation indicator of classification model can evaluate the model from the accuracy, stability, generalization ability and other aspects comprehensively.

Firstly, calculate the Davies-Bouldin index and Calinski-Harabasz index of the clustering result of K-means used in [9], FCM used in [13], and another sub-clustering algorithm [36] in proposed pre-classification model which named spectral clustering by (1)–(7). Then calculate the two indexes of the data classified by the proposed model. The results retain 4 decimal places, and the calculation results are shown in Table 11.

**Table 11:** Comparison of the values of DBI and CH indicator.

Method	Value of DBI	Value of CH
FCM [13]	0.7614	438.3526
Spectral clustering	0.7377	414.7100
K-means [9]	0.8112	316.0603
**This work**	**0.7428**	**457.8629**

It can be seen from Table 11 that in comparison with the Davies-Bouldin index and Calinski-Harabasz index of FCM clustering results in [13], the Davies-Bouldin index value of the data classified by the proposed model is lesser than the result of direct use of FCM, and the value of Calinski-Harabasz index is greater than that of direct use of FCM. This indicates that the three categories separated by the proposed model are farther apart and the samples within each category is closer together. Then, comparing the two indexes of spectral clustering and the proposed method, although the Davies-Bouldin index value of the proposed method is slightly larger than the spectral clustering, the Calinski-Harabasz index value is much larger than the it. The comprehensive comparison of the two indexes also shows that the classification results of the proposed model are more in line with the classification principle that the farther the distance between categories is the better, and the closer the distance within categories is the better. Finally, comparing the two indexes of K-means and the proposed model, the proposed method performs much better than it. Therefore, the classification results of the proposed model make the distance between the categories of data further, the distance within the categories closer, and the classification of data more reasonable and effective.

**Fig 10:**
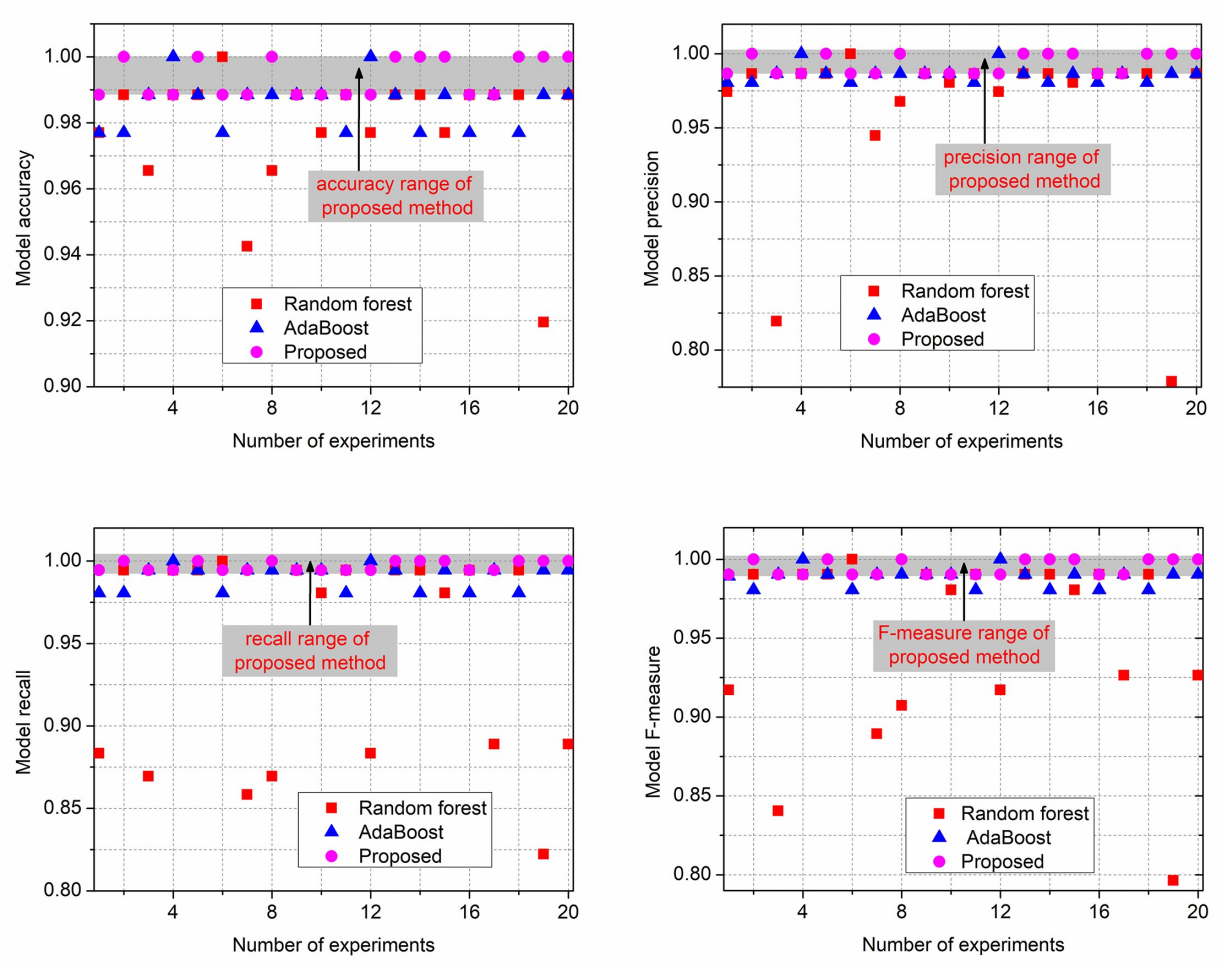
Model evaluation indicators comparison. (a) The comparison of model accuracy; (b) The comparison of model precision; (c) The comparison of model recall; (d) The comparison of model F-measure.

Secondly, compare the proposed majority voting ensemble learning method with the conventional ensemble learning method RF and AdaBoost. The number of individual classifiers of RF and AdaBoost were set to be 3, which consistent with the number of individual classifiers of proposed model, and the proposed model, RF, and AdaBoost were trained and tested for 20 times. The accuracy, recall, precision and F-measure of three models are obtained by (8)–(11). And the experiment results are shown in Fig 10, which red, blue and pink dots respectively represent the model of the RF, AdaBoost and this work. The gray areas in the figures are the range of indexes values. It can be seen that the proposed model’s accuracy, recall, precision and F-measure are more stable in 20 experiments and the value of evaluation indexes are higher than the other two methods, while training and testing on the labeled data set. The accuracy rate represents the prediction accuracy of the whole dataset, and the higher the accuracy, the closer the model classification results are to the real results. The recall rate represents the probability of actually positive samples that being predicted to be positive, and the precision rate represents the probability of being predicted to be positive that is actually positive sample, and they restrict and influence each other. F-measure is a harmonic average of precision and recall rates, which can balance the influence of precision rate and recall rate and evaluate a classifier more comprehensively. Therefore, the higher the value of these four indexes are, the better the classifier is. In addition, we can obtain that ensemble learning methods have a good effect in dealing with classification problems. The model accuracy of the three methods were all higher than 90%, and other indexes are also higher than 80%. The average value of the evaluation indexes for 20 experiments and the running time of the proposed method and the conventional ensemble learning method are presented in Table 12. The data in Table 12 is obtained by retaining the four decimal places for the actual value. As can be seen from Table 12, the running time of the proposed model is slightly higher than that of RF and AdaBoost, but the performances of the other four indexes are significantly better than them. This indicates that the proposed majority voting ensemble learning method has better classification ability and robustness than the conventional ensemble learning method. And it is more suitable for solving complex classification problems.

**Table 12:** Comparison of the average value of evaluation indicators of different ensemble learning models.

Model	Time (s)	Accuracy	Recall	Precision	F-measure
Random forest	0.3022	97.87%	94.38%	96.37%	94.98%
AdaBoost	0.3352	98.56%	99.01%	98.59%	98.83%
**This work**	**0.3581**	**99.37%**	**99.69%**	**99.27%**	**99.47%**

Finally, compare the accuracy, recall, precision and F-measure of the proposed model with the methods used in [16, 22, 23, 26, 37] for the same dataset. The specific methods and the value of indexes of these models are presented in Table 12. As can be seen from Table 13, the neural network model does not perform well in this task, possibly because the feature dataset is not large enough. SVM performs well in this task compared with neural networks. The main reason is that SVM is more sensitive to small sample dataset and can train better classifier with less training dataset. Moreover, the performance of ensemble learning method in this work is generally higher than other machine learning methods, because ensemble learning avoids the limitation of single algorithm in classification decision making and improves stability while ensuring accuracy. In addition, the method used to label has an impact on the final result. The rationality of labels directly affects the validity of subsequent classification. Therefore, the method selection of pre-classification stage is also very important. It can be seen from the table that the same classifier has different performance while uses different clustering methods, so the effectiveness of the method needs to be considered in many aspects when building the model. With a comprehensive comparison of the four indexes, it can be seen that the proposed method performs better than other methods in the Table 13, which indicates the proposed method is able to provide a reliable support for the realization of automatic driving technology, and also provides a reference for usage-based insurance.

**Table 13:** Comparison of the value of evaluation indicators of different models for the same dataset.

Ref.	clustering algorithm	classifier	Accuracy	Recall	Precision	F-measure
[16]	FCM	BPN	81.25%	60.74%	55.56%	56.75%
[22]	K-means	SVM	98.89%	98.61%	99.49%	99.03%
[23]	PCA+FCM	SVM	98.89%	98%	99.24%	98.60%
[26]	K-means	AdaBoost	98.89%	99.49%	98.61%	99.04%
[37]	K-means	BPN	75%	26.09%	33.33%	29.27%
**This work**	**FCM+SC**	**SVM+KNN+CART**	**99.20%**	**99.61%**	**99.07%**	**99.33%**

## Conclusion

Based on the internet of vehicles data of 450 transport vehicles from the competition platform, this paper extracts and quantifies the parameters of driving behavior features, and comprehensively uses various classification methods to deal with the problem. The model consists of pre-classification stage and classification stage, and the ensemble learning is used in both stages. In the pre-classification, an ensembled clustering method based on FCM and spectral clustering is used to cluster the driving behavior feature parameters, thereby the data are divided into labeled dataset and unclassified dataset. With the results of pre-classification, a majority voting ensemble learning classification method based on CART, SVM, and KNN is trained and tested. A variety of individual classifiers are used for learning, and a majority voting strategy is used to combine the three individual classifiers, and thus classify the driving style of vehicles.

A correlation mechanism of internet of vehicles data, driving behavior characteristics and traffic safety is established by the classification of driving styles and mining of drivers’ driving behavior habits, which provides a reference for the transportation management department to monitor and assess drivers. At the same time, this paper improves the generalization ability, stability and accuracy of driving behavior classification model through the combination of various individual classifiers. However, there are some limitations in this paper, for example, the data dimension is not rich enough, which does not include road information and vehicle condition information. More factors should be considered in future researches. Factors such as weather and road conditions can be taken into account, and more diverse data can make the final evaluation more accurate and complete. What’s more, automatic feature screening can also be studied in future.
